# Supplementary material for: Determination of Pseudomonas aeruginosa MexXY-OprM substrate profile in a major efflux knockout system reveals distinct antibiotic substrate classes
Source: Microbiol Spectr. 2025 Feb 6;13(3):e02903-24. doi: 10.1128/spectrum.02903-24 (PMC11878066; doi:10.1128/spectrum.02903-24)
Supplement: Supplemental figures and tables — Fig. S1 and S2; Tables S1 to S4. [file spectrum.02903-24-s0001.pdf]

SUPPLEMENTAL MATERIAL

Determination of *Pseudomonas aeruginosa* MexXY-OprM specificity in a major efflux knockout system reveals distinct antibiotic substrate classes

Logan G. Kavanaugh[1,2], Shraddha M. Hariharan[1], and Graeme L. Conn[1,2,3]

[1]Department of Biochemistry, Emory University School of Medicine, Atlanta, GA

[2]Microbiology and Molecular Genetics Graduate Program, Emory University, Atlanta, GA

[3]Emory Antibiotic Resistance Center, Emory University, Atlanta, GA

Address correspondence to: gconn@emory.edu

Running Title: MexXY-OprM substrate specificity

Key Words: Efflux, *Pseudomonas,* antimicrobial susceptibility, antimicrobial resistance, resistance-nodulation-division (RND)

This file contains:

Supplemental Figures:

Figure S1. Rhamnose induction assay for MexXY expression and cell viability
Figure S2. MexY expression in strain LK21

Supplemental Tables:

Table S1. Strains and plasmids used in this study
Table S2. DNA primers used in this study
Table S3. Gentamicin minimum inhibitory concentration (MIC) in LK21 in the presence of 1% arabinose and variable rhamnose concentrations for MexXY expression
Table S4. Antimicrobial susceptibility data for *P. aeruginosa* PAO1 and PΔ6 expressing MexXY, OprM, or the whole efflux pump MexXY-OprM.

Supplemental Figures

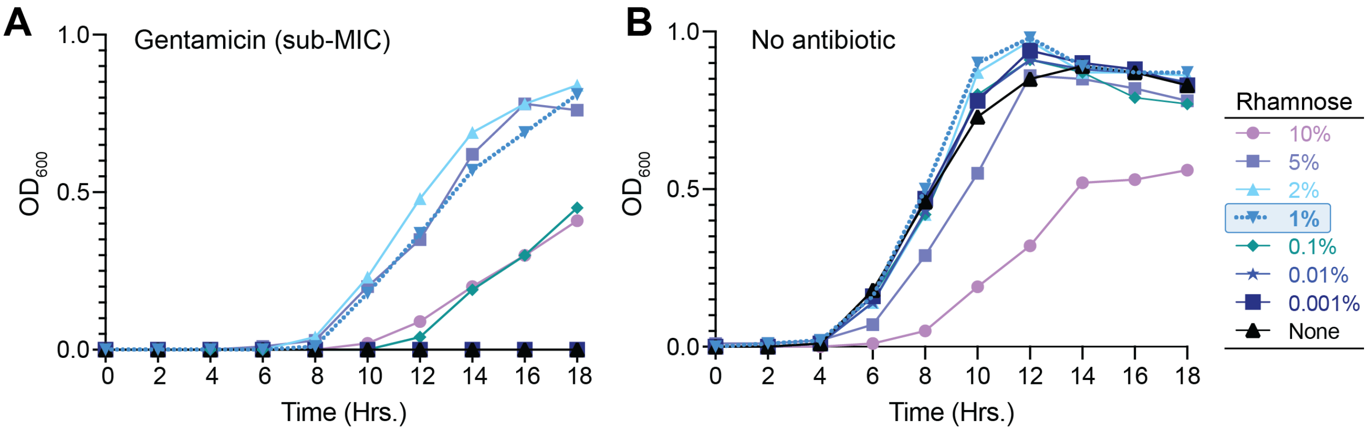

Fig. S1. Rhamnose induction assay for MexXY expression and cell viability. Optical density at 600 nm (OD_{600}) of strain LK21 grown over 18 hours in cation-adjusted Mueller-Hinton broth supplemented with 1% arabinose ***A***, at the greatest subinhibitory concentration of gentamicin (8 µg/mL) and ***B***, in the absence of antibiotic at variable concentrations (0.001-10%) of rhamnose. The no rhamnose control is shown in black diamond and the selected inducer concentration (1%) is highlighted with the blue dotted line.

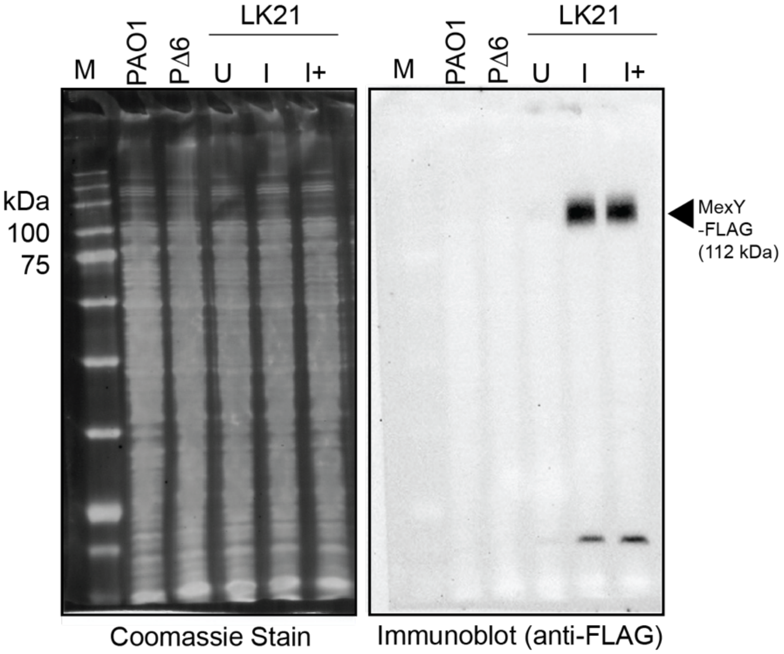

Fig. S2. MexY expression in strain LK21. Coomassie stained SDS-PAGE gel (*left*) and HRP-conjugated anti-FLAG (ABclonal) immunoblot used to detect the C-terminal MexY FLAG (*right*). Lanes shown are whole cell lysate for PAO1, PΔ6, and LK21 with no inducer (U), MexXY induced with 1% wt/vol rhamnose (I), or MexXY and OprM induced with 1% wt/vol rhamnose and arabinose (I+). MexY-FLAG (112 kDa) migrated the appropriate size shown by protein marker (M).

Supplemental Tables

Table S1. Strains and plasmids used in this study.

Strain	Genotype	Reference
PAO1	*P. aeruginosa* laboratory strain	Goldberg Lab
PΔ6	PAO1 Δ*mexAB-oprM,* Δ*mexCD-oprJ,* Δ*mexEF-oprN,* Δ*mexJKL,* Δ*mexXY,* Δ*triABC*	1,2
LK17	PΔ6::*mexXY*^{PAO1}	This study
LK21	PΔ6::P$_{rha}$-rbs-*mexXY*^{PAO1}, pHERD20T-P$_{BAD}$-*oprM*^{PAO1}	This study
LK36	PΔ6, pHERD20T- P$_{BAD}$-*oprM*^{PAO1}	This study
**Plasmid**	**Genotype**	**Reference**
pJM220	pUC18-miniTn7T-gm-rhaSR-P$_{rha}$BAD	3
pTn7XY	pUC18-miniTn7T-gm-rhaSR-PrhaBAD-*rbs_mexXY*^{PAO1}	This study
pTn7XY-FLAG	pUC18-miniTn7T-gm-rhaSR-PrhaBAD-*rbs_mexXY*^{PAO1}*-FLAG*	This study
pHERD20T	pUCP20T-araC-P$_{BAD}$	4
pHD20TM	pUCP20T-araC-P$_{BAD}$-*oprM*^{PAO1}	This study

Table S2. DNA primers used in this study.

Primer name	Sequence (5’-3’)
PAO1_HindIII_RBS*mexX-F*	GCGAAGCTTTGAACGTCCTCACAAGGGAAAG
PAO1_ApaI_*mexY-R*	GCGGGGCCCTCAGGCTTGCTCCGTG
PAO1_EcoRI_*oprM-F*	GCGGAATTCATGAAACGGTCCTTCCTTTC
PAO1_HindIII_*oprM-R*	GCGAAGCTTTCAAGCCTGGGGATCTTC
MexY_FLAG-F	GACTACAAGGACGACGATGACAAGTGACTCGCGAAGGCC
MexY_FLAG-R	GGCTTGCTCCGTG

Table S3. Gentamicin minimum inhibitory concentration (MIC) in LK21 in the presence of 1% arabinose and variable rhamnose concentrations for MexXY expression.

		Minimum inhibitory concentration (µg/mL)							
Inducer	***% Rha***	*10*	*5*	*2*	*1*	*0.1*	*0.01*	*0.001*	*0*
Antibiotic	Gen	16	16	16	16	16	8	8	2

Table S4. Antimicrobial susceptibility data for *P. aeruginosa* PAO1 and P∆6 expressing MexXY, OprM, or the whole efflux pump MexXY-OprM.

		Minimum inhibitory concentrations (µg/mL)[a]						
		**PAO1**		**P∆6**		**LK17**	**LK36**	**LK21**
	Genotype	WT		∆ABM/ CDJ/ EFN/ JKL/ XY/ triABC		P∆6 + XY	P∆6 + M	P∆6 + XYM
Inducer	*rha*	-	+	-	+	+	-	+
	ara	-	+	-	+	-	+	+
Aminoglycosides	Kan	64	64	16	32	32	32	128
	Amk	1	2	0.5	1	2	1	16
	Gen	2	4	0.5	2	2	1	16
	Tob	1	2	1	2	2	2	8
	Neo	16	32	8	16	16	16	64
	Par	64	128	8	16	16	32	>128
Tetracycline	Tgc	4	4	0.5	0.5	1	0.5	8
Macrolide	Ery	128	>128	4	4	16	16	>128
Fluoroquinolone	Cip	0.25	0.125	<0.03	<0.03	<0.03	<0.03	0.25
Dye	EtBr	>128	>128	32	32	32	32	128
Cephalosporin	Fep	0.25	0.5	0.06	0.06	0.06	0.125	2
	Caz	2	2	2	2	2	2	4
Chloramphenicol	Chl	8	16	0.5	0.5	1	1	4
Carbapenem	Mem	0.5	0.5	0.25	0.25	0.06	0.25	0.5
	Imi	4	4	4	4	4	4	8
Trimethoprim	Tmp	32	32	0.5	1	0.5	1	32
Polymyxins	Pmb	0.5	0.5	0.5	0.5	0.5	0.5	0.5

[a]Antimicrobials: kanamycin (Kan); amikacin (Amk); gentamicin (Gen); tobramycin (Tob); neomycin (Neo); paromomycin (Par); tigecycline (Tig); erythromycin (Ery); ciprofloxacin (Cip); ethidium bromide (EtBr); cefepime (Fep); ceftazidime (Caz); chloramphenicol (Chl); (Mem); imipenem (Imi); polymyxin B (Pmb); trimethoprim (Tmp)

References

1. Cooper CJ, Krishnamoorthy G, Wolloscheck D, Walker JK, Rybenkov VV, Parks JM, Zgurskaya HI. Molecular Properties That Define the Activities of Antibiotics in Escherichia coli and Pseudomonas aeruginosa. *ACS Infect Dis*. 2018;**4**(8):1223-34. Epub 20180525. doi: 10.1021/acsinfecdis.8b00036.

2. Wolloscheck D, Krishnamoorthy G, Nguyen J, Zgurskaya HI. Kinetic Control of Quorum Sensing in Pseudomonas aeruginosa by Multidrug Efflux Pumps. *ACS Infect Dis*. 2018;**4**(2):185-95. Epub 20171110. doi: 10.1021/acsinfecdis.7b00160.

3. Meisner J, Goldberg JB. The Escherichia coli rhaSR-PrhaBAD Inducible Promoter System Allows Tightly Controlled Gene Expression over a Wide Range in *Pseudomonas aeruginosa*. *Appl Environ Microbiol*. 2016;**82**(22):6715-27. Epub 2016/10/30. doi: 10.1128/aem.02041-16.

4. Qiu D, Damron FH, Mima T, Schweizer HP, Yu HD. PBAD-based shuttle vectors for functional analysis of toxic and highly regulated genes in Pseudomonas and Burkholderia spp. and other bacteria. *Appl Environ Microbiol*. 2008;**74**(23):7422-6. Epub 20081010. doi: 10.1128/aem.01369-08
